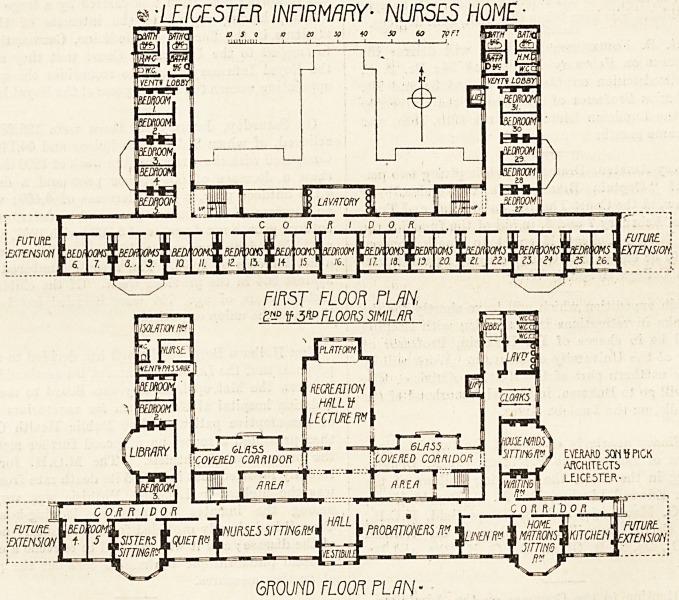# Leicester Infirmary Nurses' Home

**Published:** 1910-02-05

**Authors:** 


### LEICESTER INFIRMARY NURSES' HOME.

This building, which will be opened on Tuesday next, the 8th inst., at 3 o'clock, is planned roughly in the form of an E, the upright stroke being produced beyond the arms. The long front faces due south, the arms east and west respectively; by arranging the rooms on one side of the corridors only they are all so placed that each gets sunlight during some portion of the day. The value of this is great, but it entails a considerable addition to the length of corridor.

The main entrance is on the south. To right and left of the entrance hall are large sitting-rooms—one for the probationers, the other for staff nurses. Beyond the hall is a glass-roofed vestibule with glazed corridors on either hand. The vestibule with the side corridors is intended to be used as a winter garden. At the back, immediately facing the main entrance, is a large recreation and lecture hall provided with a stage at the north end. This room is 40 feet by 24 feet and 16 feet high; it is floored with Austrian oak, and is well adapted, not only for lectures, but for the lighter form of social gatherings. To the right of the probationers' sitting-room is the linen room, home matron's sitting-room, and kitchen; and to the left of the staff nurses' sitting-room is a class-room, sisters' sitting-room, and two bedrooms. The west wing contains a large library, three bedrooms, and an isolation ward. The latter is intended for a case of infectious illness, and is provided with a nurses' room and a w.c. The accommodation is hardly adequate for complete isolation. A small pantry and suitable provision for emptying and cleansing bed-pans would certainly be necessary, and the sanitary offices should be properly cut off from the ward. As it is, the door of the ward and that of the w.c. are within a few inches of each other. A ventilated passage separates the ward and its adjuncts from the rest of the building, and direct access to the outside is provided by a door at one end of the passage. The east wing contains a waiting-room, sitting-room for housemaids, a large cloak-room, a lavatory, and three w.c.'s. The upper floors, of which there are three, are all alike, and contain bedrooms and sanitary offices at the north ends of each wing, with a large lavatory in the middle of the main block. The total accommodation is 100 beds, including, we presume, some for servants. Each bedroom is provided with a fireplace; the floor is laid with "Stonwood," a plastic, jointless material largely composed of wood flour or finely ground sawdust. The construction generally is of fire-resisting materials. An electric lift worked on the automatic system is provided in the east wing to serve all floors.

The home, which is planned on generous lines, should prove a most valuable addition to the hospital. The work has been planned and carried out by Messrs. Everard, Son and Pick, of Leicester, the builders being Messrs. Henry Herbert and Sons. The cost of the buildings, with the furnishing, is estimated at £22,500.

---

Owing to the floods in Paris 400 patients have been removed from the Boucicaut Hospital, in the Rue de La Convention, in the Javel quarter. Wrapped in blankets and escorted by the fire brigade, these unfortunate people were conveyed in motor cars to infirmaries in other parts of the city. Fears were entertained, says the *Times* correspondent, for the regularity of the food supply of hospitals and other public institutions.

## Figures and Tables

**Figure f1:**